# Effect of Hemp Fiber Surface Treatment on the Moisture/Water Resistance and Reaction to Fire of Reinforced PLA Composites

**DOI:** 10.3390/ma14154332

**Published:** 2021-08-03

**Authors:** Percy Festus Alao, Laetitia Marrot, Heikko Kallakas, Alar Just, Triinu Poltimäe, Jaan Kers

**Affiliations:** 1 Department of Material and Environmental Technology, Tallinn University of Technology, Ehitajate tee 5, 19086 Tallinn, Estonia; heikko.kallakas@taltech.ee (H.K.); triinu.poltimae@taltech.ee (T.P.); jaan.kers@taltech.ee (J.K.); 2 InnoRenew CoE, Livade 6, 6310 Izola, Slovenia; laetitia.marrot@innorenew.eu; 3 Department of Civil Engineering and Architecture, Tallinn University of Technology, Ehitajate tee 5, 19086 Tallinn, Estonia; alar.just@taltech.ee

**Keywords:** hemp fiber composites, water absorption, hygroscopic properties, thermal properties, fire resistance

## Abstract

The effects of surface pretreatment (water and alkali) and modification with silane on moisture sorption, water resistance, and reaction to fire of hemp fiber reinforced polylactic acid (PLA) composites at two fiber loading contents (30 and 50 wt.%) are investigated in this work. Moisture adsorption was evaluated at 30, 50, 75 and 95% relative humidity, and water resistance was determined after a 28-day immersion period. The cone calorimetry technique was used to investigate response to fire. The fiber surface treatment resulted in the removal of cell wall components, which increased fiber individualization and homogeneity as shown in scanning microscopic pictures of the composite cross-section. Although the improved fiber/matrix bonding increased the composite’s water resistance, the different fiber treatments generated equal moisture adsorption results for the 30 wt.% reinforced composites. Overall, increasing the fiber amount from 30 to 50 wt.% increased the composite sensitivity to moisture/water, mainly due to the availability of more hydroxyl groups and to the development of a higher pore volume, but fire protection improved due to a reduction in the rate of thermal degradation induced by the reduced PLA content. The new Oswin’s model predicted the composite adsorption isotherm well. The 30 wt.% alkali and silane treated hemp fiber composite had the lowest overall adsorption (9%) while the 50 wt.% variant produced the highest ignition temperature (181 ± 18 °C).

## 1. Introduction

The EU Green deal policy aims to achieve a carbon neutral economy by 2050, which requires redesigning different industrial sectors, their processes and their products. As a result, there is a growing challenge toward the use of healthy, sustainable, non-hazardous, biodegradable and renewable resources while maintaining reliability in product performances [1]. Due to low embodied production energy, plant fibers have gained a lot of attention in this regard [2,3]. Flax, jute, hemp, sisal and ramie figure amongst the widely accessible source of plant fibers [4]. Specifically, studies [5,6,7,8] have shown that hemp fiber can be a suitable natural reinforcing material for composite application due to its vast availability, price stability and good mechanical properties.

Furthermore, to obtain fully compostable composites and to enhance positive environmental impact, bio-thermoplastic matrices such as polylactic acid (PLA) are being preferred to replace traditional synthetic polymers with the same performances (polyester, polypropylene and polyurethane). PLA is produced from annual bioresources, mainly through the fermentation of corn, and display a cost-effective production compared to other polymers developed from renewable resources such as soya oil-based epoxy, lignin and starch [9]. Hemp PLA composites have shown promising results [10] and can be regarded as appropriate materials for a vast range of applications and minimize the environmental threats associated with petroleum-based products.

Despite their proven competitive properties when compared to composites reinforced with synthetic fibers, there is a crucial need to improve performance and durability during service of natural fiber composites. The performance of natural fibers as a reinforcing material is influenced by essential parameters such as fiber treatment and content, matrix/binder type, interfacial strength and the manufacturing process [11]. Natural fibers are composed of cellulose and amorphous cell wall biopolymers (hemicellulose, pectins and lignin), highly rich in hydroxyl groups responsible for the hydrophilic nature of the fiber and conferring a high affinity for moisture. When these fibers are blended with the hydrophobic matrix, weak interfacial bonds are formed, spawning a deterioration in service due to voids and debonding within the composite [5]. The hemicellulose and lignin covering components reduce the ability of cellulose, which is liable for the specific mechanical properties and structural stability, to effectively adhere to the polymer matrix. The hygroscopic properties of such plant-based composite materials are thus affected by the high moisture sensitivity resulting from the presence of these hydroxyl groups [2]. The chemical modification of the fibers appears to successfully remove the covering materials (hemicellulose, lignin & extractives) responsible for the moisture absorption [12,13]. According to Pejic et al. [14] the alkali treatment of hemp fibers results in the gradual removal of hemicellulose and lignin that causes the rearrangement of fibrils and the formation of new capillary spaces between completely or partially separated fibers, which increases the roughness of the hemp fiber surface and enhances wetting with the polymer matrix. Additionally, the modification of the hemp fiber surface by alkali treatment brought improvements of the composite tensile and flexural properties [10]. Due to their elemental composition (carbon, hydrogen and oxygen) associated with cellulose, hemicelluloses and lignin main components, lignocellulosic fibers are flammable and can easily decompose in the event of a fire hazard [15]. The fire resistance characterization of hemp fiber reinforced polyesters was assessed by Naughton et al. [16]. It was reported that the increase in the composite hemp fiber volume contributed to the formation of an effective thermally insulating char layer. However, based on current knowledge, there is no recent study about the fire behavior of hemp fiber reinforced PLA composites and the effect of surface treatments on the composite fire reaction. The characterization of the fire resistance would increase the consideration of natural fiber reinforced composites in construction and demanding transportation (automobile/aeronautical) applications. 

This study focused on inspecting the contribution of surface pretreatment (water and alkali pretreatment) and modification (silane treatment) on the moisture adsorption, water absorption and reaction to fire of hemp fiber reinforced PLA (HPLA) composites. The objectives of this work are as follows:

i To produce composites with two fiber loadings (30 and 50 wt.%) using frost-retted hemp fibers from Estonia and polylactic acid (PLA). These locally obtained hemp fibers are commonly considered as waste. Using them as PLA reinforcement is a way to value these by-products from the cannabidiol industry [17] and to enhance the contribution to a carbon-neutral environment;
ii To study the influence of a combination of fiber surface pretreatments and modification of hemp fibers on the hemp PLA properties of interest (moisture/water resistance and fire behaviour) to promote the development of biocomposites as building materials.

**Figure 1:**
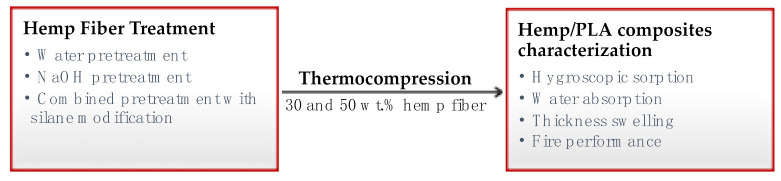
Schematic representation of the research objective.

Figure 1 presents a scheme of the main objective of this research.

## 2. Materials and Methods

### 2.1. Materials

The hemp fibers used for this study were grown, frost-retted and harvested between 10th of June 2016 and 4th of May 2017, in Saaremaa, Estonia. The chemical composition of these hemp fibers has been described previously in our paper [17] and is reported in Table 1. The fibers were manually cleaned to remove hurds, carded twice with a wide classic drum carder (300 mm batt width and 72 teeth per inch) and dried in the oven at 80 °C to achieve uniform weight before use. Staple polylactic acid (PLA) from NatureWorks LLC was used as a matrix. The density of the PLA was 1.24 g.cm^−3^.

### 2.2. Methods

#### 2.2.1. Hemp Fibers Treatments

**Figure 2:**
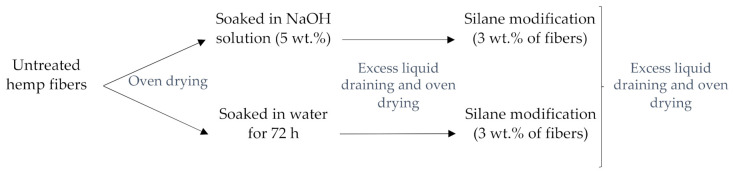
The surface pretreatments and treatment of hemp fibers.

The hemp fiber pretreatments (water, alkali) and modification with silane were carried out according to Alao et al. [10] (Figure 2). In all cases, the hemp fibers were oven-dried at 80 °C before and after treatment. The pretreatment with 5 wt.% NaOH was performed for 4 h at room temperature (23 °C). Regarding the silane treatments, the hemp fibers (water/alkali pretreated) were soaked in a solution of 3-Aminopropyl-triethoxy silane (3 wt.% of hemp fibers) with 99% concentration and ethanol (97% concentration, 80 vol.%) with distilled water (20 vol.%) for 2 h. Before the treatment process, the solution was activated with acetic acid and constantly stirred for 2 h to control the pH to 5 and to pre-hydrolyze the silane.

Hemp fibers are pretreated to eliminate non-cellulosic compounds (hemicellulose, lignin and pectins). The elimination of these components results in a loss of fiber weight. The fiber weight reduction (*F_W_*) associated to each treatment was measured on three batches of 10 g hemp fibers and was estimated using the following Equation (1):

(1) $$F_{w}\;\left(\%\right)=\frac{F_{0}-F}{F_{0}}\times 100$$

where *F_0_* is the weight of the fiber before any treatment, and *F* is the weight of the fiber after pretreatment/treatment.

#### 2.2.2. Fabrication of the Hemp Reinforced Polylactide (HPLA) Composite

**Figure 3:**
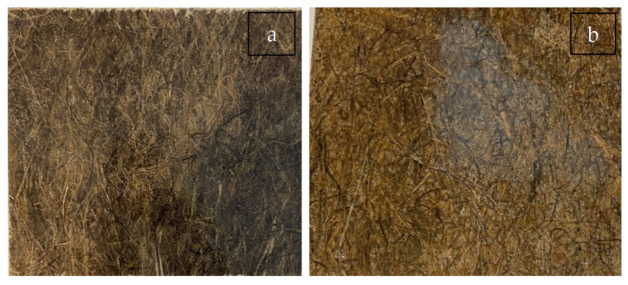
Specimens (**a**) 30 wt.% hemp reinforced PLA composite and (**b**) 50 wt.% hemp reinforced PLA composite.

The dried untreated and treated hemp fibers were combined with PLA fibers by hot pressing. Ten composite variants were produced based on hemp fiber composition (30 wt.%; 50 wt.%) and treatment type (UH: untreated; WH: water treated; WSH: water + silane; AH: alkali treated; ASH: alkali + silane). Hemp and PLA fibers were weighed using a ±0.1 mg precision balance (Mettler Toledo ME4002E, Greifensee, Switzerland) and carded. The mix was transferred to a metal frame (450 mm × 450 mm × 2 mm), then pre-heated for 5 min at a temperature of 180 °C before applying a pressure of 3 MPa for 10 min. The final product was allowed to cure at room temperature for 30 min underweight before removal from the frame. A silicon-based release agent was used to prevent sticking of the fabricated composite to the mould. Figure 3 shows the hemp reinforced PLA composite from untreated hemp fiber at Figure 3a 30 wt.% and Figure 3b 50 wt.%.

The composite void fraction (*V_p_* %) was calculated using Equation (2) according to Marrot et al. [17] from the density of the fibrous reinforcement (ρ*_f_*) (taken as 1.38 gcm^−3^), density of the PLA matrix (ρ*_m_*) (1.24 gcm^−3^) and density of the composites (ρ*_c_*).

(2) $$V_{P}\left(\%\right)=1-\frac{\frac{W_{f}}{\rho_{f}}+\frac{W_{m}}{\rho_{m}}}{\frac{W_{c}}{\rho_{c}}}\times 100$$

where *W_f_*, *W_m_* and *W_c_* represent the weight of the hemp fiber, PLA and the composite, respectively.

#### 2.2.3. Scanning Electron Microscopy (SEM)

The morphology of the composite cross section was studied using a Zeiss Ultra v.55 (FELMIZFE, Graz, Austria) SEM at 20 kV, depth of 100 μm and 50,000 resolution. The samples were mounted into epoxy glue and coated with 2 nm thick gold-palladium layer (80% Au + 20% Pd) before imaging.

#### 2.2.4. Moisture Adsorption Properties

The moisture sorption was performed following the EN ISO 12571:2013 standard at relative humidity (RH) levels of 30, 50, 75 and 95% and constant temperature (23 ± 0.5 °C) in a climatic chamber. Four replicas of each composite variant measuring 100 mm × 100 mm were dried in the oven as stipulated by EN ISO 12570:2000 standard and weighed until the mass change was ≤0.1% of the preceding 24 h. The data for the equilibrium moisture content (*EMC*) was obtained by weighing the samples at the specified RH until the weight was ≤0.1% of the previous 24 h. The *EMC* on dry bases (d.b) was then calculated using the following Equation (3). The obtained *EMC*s were statistically analyzed with ANOVA single factor and an alpha of 0.05.

(3) $$EMC_{d.b}\left(\%\right)=\frac{M-M_{0}}{M_{0}}\times 100$$

where *M_0_* is the mass of the oven dried samples; *M* is the mass of the specimens at any given RH. 

The modified Oswin model shown in Equation (4), identified by Palumbo et al. [18] as a good fitting model, was used to predict the moisture adsorption. This model has also been adopted for frost-retted hemp fibers by Nilsson et al. [19].

(4) $$M_{D}=\frac{\left(A+\mathrm{BT}\right)}{{\left(H_{R}-1\right)}^{\frac{1}{C}}}\;$$

where *M_D_* is equilibrium moisture content, d.b%; *H_R_* is the relative humidity in decimal; *T* is the temperature, °C; *A*, *B* and *C* are the modified Oswin model constants.

The parameters in the equation were determined using nonlinear regression analysis. In this regard, constants *A*, *B* and *C* were assumed values and then used to calculate the model (*M_D_*) at the experimental temperature and the specific relative humidity. For each specimen, the differences between the measured equilibrium moisture contents (*EMC*_d.b_) and the estimated equilibrium moisture contents (*M_D_*) were taken for each RH to obtain the sum of squares. This sum of squares was then reapplied in a non-linear regression to re-obtain the value of the constants *A*, *B* and *C*, which were then used in the model fitting.

#### 2.2.5. Long-Term Water Absorption (WA) and Thickness Swelling (TS)

The long-term WA was determined by measuring the mass change of the specimen after an immersion in water for 28 days in accordance with EN ISO 16535 standard from five replicas that were first conditioned at 23  ± 2  °C and RH of 50  ± 5% for 24 h. The TS was determined based on the EN 317 standard after 28 days of water immersion. The initial thickness of the specimens was measured following the EN 325 standard using a digital micrometer screw gauge (Hans Schmidt & Co. GmbH, Waldkraiburg, Germany) with a precision of ± 0.01 mm. All specimens were conditioned in the climatic chamber at a temperature of 20  ± 2  °C and RH of 65  ± 5% before the test. For the test, specimens were placed uprightly in water covering up to 25 ± 5 mm of the top edges. The *WA* (Equation (5)) and *TS* (Equation (6)) were calculated and analyzed with ANOVA single way using an alpha of 0.05.

(5) $$WA\;\left(\%\right)=\frac{W-W_{0}}{W_{0}}\times 100$$

where *W_0_* is the mass of the conditioned specimens (g) prior to water immersion; *W* is the mass of the specimens at the end of 28 days of immersion.

(6) $$TS\;\left(\%\right)=\frac{T-T_{0}}{T_{0}}\times 100TS\;\left(\%\right)=\frac{T-T_{0}}{T_{0}}\times 100$$

where *T_0_* is thickness of the conditioned specimens (mm) prior to water immersion; *T* is the thickness of the specimens at the end of 28 days of immersion.

#### 2.2.6. Reaction to Fire of the Composites

**Figure 4:**
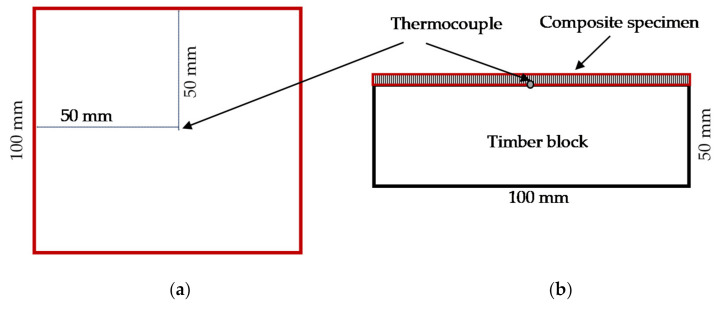
The set-up of test specimen: (**a**) top view of timber surface; (**b**) cross-sectional view of specimen on timber block.

The cone calorimeter was used to investigate the fire test in accordance with the EN 5660-1:2015 standard. This approach was chosen because it is useful for estimating the fire safety of materials in European classification [20]. Composite samples (100 × 100 mm^2^ in size) were exposed to a heat flux of 50 kWm^−2^ using a cone heater. The specimens were conditioned for seven (7) days prior to the test at a temperature of 23 °C and RH of 50%. Figure 4 depicts a 2D drawing of the specimen setup on a timber (pine) block. Timber was used as base material because it is an important construction material susceptible to fire hazards. The hemp PLA composite is assessed as a protection against the thermal degradation of the timber, though the research disregarded the decomposition of the timber. To ensure uniformity of results, all timber specimens were cut from the same pine lumber and conditioned with the same parameters as the hemp PLA composite prior to the experiment, as stipulated in the standard. A 0.25 mm diameter type K thermocouple (Pentronic AB, Vastervik, Sweden) was placed at the midpoint (50 mm) on the top of the specimen, to record the surface temperature, and between the composite and timber block. The edge of the entire piece was covered with self-bonding aluminium tape that reached 0.5 mm on the sample surface. The specimen was mounted in a retainer frame with the exposed surface held at a distance of 60 mm from the base of the cone heater. The result of the fire test is shown as a temperature-time curve at 5 s intervals. To correctly predict the composites’ fire reaction, the mass and thickness of the composites were measured. The composite fire performance was characterized in terms of (i) Ignition time, (ii) Ignition temperature, (iii) Temperature response through depth (i.e., temperature determined between the surface of the composite and the timber block), (iv) Weight loss, (v) Basic protection time of the composite (t_prot_) and (vi) Start time of charring of timber (t_ch_), which is a function of t_prot_ of the composites. Temperature values of 270 °C and 300 °C were used to obtain the t_prot_ and t_ch_, respectively, as recommended in the literature [21].

## 3. Results and Discussion

### 3.1. Impact of Fiber Treatment on the Fiber Mass

Table 2 presents the hemp fiber mass change following the pretreatments/treatment. For both pretreatments, hemp fibers showed a reduction in weight. The 4% weight reduction measured on the water pretreated hemp fibers is the result of the removal of water-soluble, fiber cell wall contents, as it was qualitatively characterized by Fourier transform infrared (FTIR) in a previous work [10] and also observed by Bourmoud et al. [22]. The higher 14% drop in fiber mass due to alkali pretreatment is attributable to the extraction of significant non-cellulosic components (hemicelluloses, lignin, and some quantities of solubles (wax)) [10]. Indeed, in a previous study, FTIR peaks attributed to conjugated carboxylic esters in hemicelluloses and wax, acetyl groups in lignin and conjugated carbonyl groups in lignin disappeared after alkali pretreatment [10]. The overall quantity of non-cellulosic content in the hemp fiber used in this study is 22.6 ± 0.7%, including 8.3% hemicellulose and 12.6% solubles (shown in Table 1), suggesting that the hemp fiber still contains some non-cellulosic components after pretreatments. Hu et al. [23] reported 25.9% weight reduction of hemp fibers after treatment with 6% NaOH solution at 40 °C for 24 h. The extended treatment duration and the volume of NaOH solution used by Hu et al. [23] possibly explain the discrepancy in results. However, no information was presented about fiber biochemical composition in the cited study.

The outcome for the water pretreated fibers following silane treatment show that when significant amounts of non-cellulosic fiber content are present, portions of the hemicellulose can be removed in addition to the deposition of silane molecules on the hemp fiber surface. These non-cellulosic fiber components may have been extracted during the hemp fiber immersion in the ethanol/water solution. Regarding the combined alkali and silane treatment, the hemp fibers showed a 0.9% weight gain, which can be attributed to the surface deposition of silane molecules.

### 3.2. Cross-Sectional SEM Observations of the Hemp Reinforced PLA Composites

**Figure 5:**
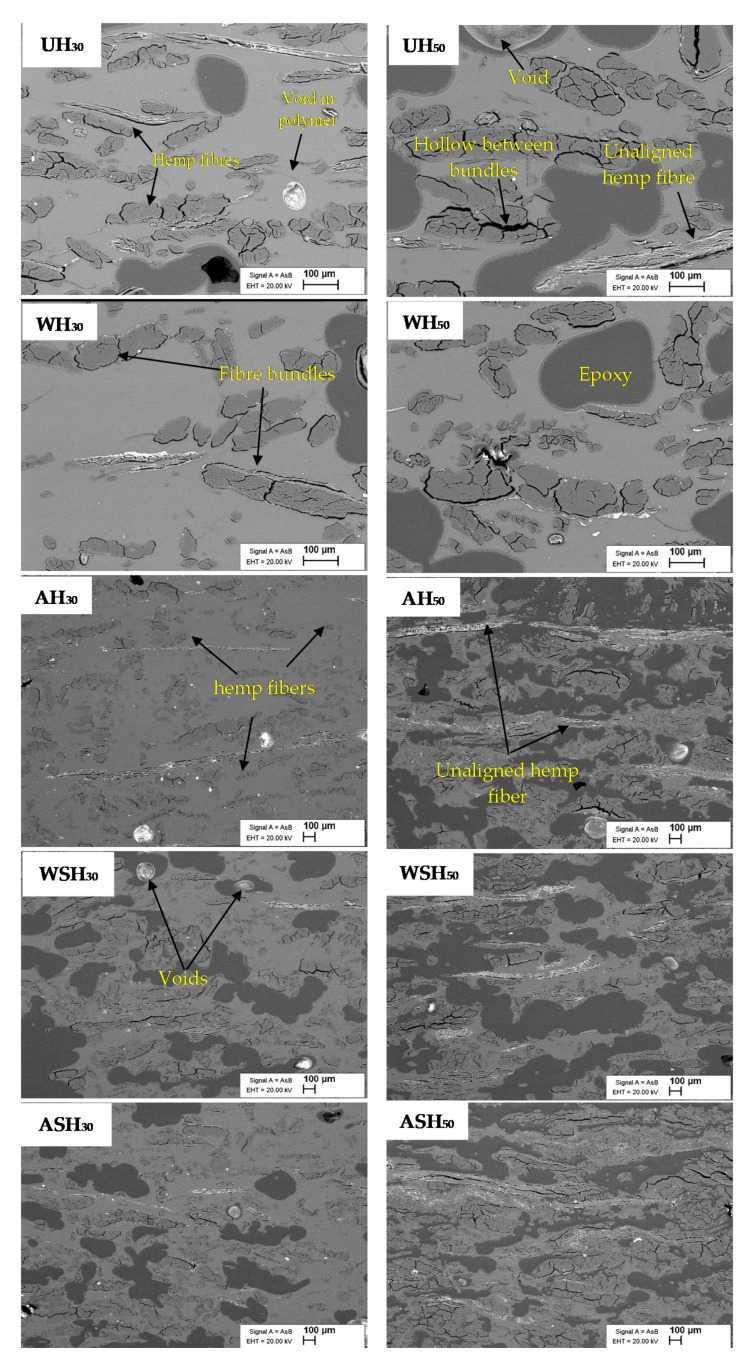
The cross-sectional images of reinforced PLA composites (UH, WH, AH, WSH and ASH) at 30 and 50% hemp fiber loading.

The cross-sectional SEM images of the hemp fiber reinforced PLA composites are shown in Figure 5 for the untreated (UH_30_; UH_50_), water pretreated (WH_30_; WH_50_), alkali pretreated (AH_30_; AH_50_), water-silane treated (WSH_30_; WSH_50_) and alkali-silane treated (ASH_30_; ASH_50_) hemp fiber reinforced composites at 30 and 50 wt.%. The darker spots observed in the SEM images and identified on WH_50_ is related to the epoxy glue used to embed all the composites before the imaging. Some voids can be observed in most of the specimens, which suggest air entrapment within the composites during the fabrication process. The volume of porosities *V_p_* of the composites are presented in Table 3. For both fiber contents, the volume of porosities was the highest for UH samples, due to the high amount of non-cellulosic components at the surface of the hemp fibers which prevents the wetting of the fibers by the PLA matrix, in addition to the weak interfacial bonds formed between fibers and the hydrophobic matrix. The removal of non-cellulosic components and the coupling brought by the silane agent tended to decrease the volume of porosities. Composites with 50 wt.% fibers displayed higher volumes of porosities overall when compared to 30 wt.% fibers, due to the additional difficulties in wetting and matrix flow during processing linked to the increased fiber amount.

UH composites are characterized by poorly dispersed, aggregated fiber bundles having inherent defibrillations. WH composites show a similar outcome, though fiber aggregates are more homogeneous and fewer bundles are visible, especially for 50 wt.% fiber content. The removal of water-soluble, fiber cell wall contents induced by the water pretreatment contributed to the better fiber individualization and homogeneity within the composite network without discernible effect on the surface morphology. The AH composites cross-sections appear homogenous, with individualized and uniformly dispersed fibers within the PLA matrix. Ray et al. [24] found that alkali fiber pretreatment causes gaps in the fiber structure owing to the extraction of fiber binding components, splitting the strands into single fibers and improving dispersion.

When WSH is compared to WH composites, the silane treatment resulted in clearer improvements in fiber separation, homogeneity and dispersion, which may be partly attributed to the elimination of a portion of the cell wall component, as seen in Table 2. Though ASH composites show similar observation as AH composites at 30% fiber loading, at 50 wt.% the composites present more homogenous fibers and fewer voids. As previously reported [10], there is a poor fiber wetting by the PLA matrix at 50 wt.% fiber content. Hence, the effective condensation of the siloxane layer on the fiber surface following silane modification improved the fiber/matrix adhesion more visibly at the higher fiber content.

### 3.3. Moisture Adsorption Properties

**Figure 6:**
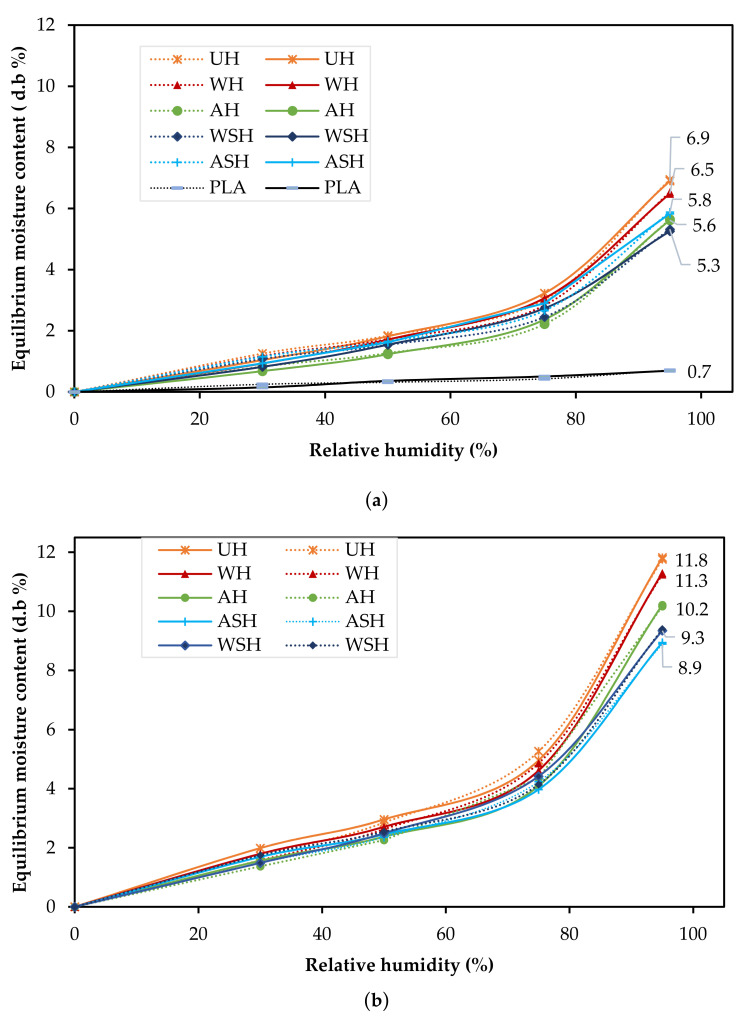
Adsorption isotherms (predicted (- -) and observed (—) for (**a**) the neat PLA, the 30wt.% composites and (**b**) the 50 wt.% composites (untreated (UH ж), water pretreated (WH ▲), alkali pretreated (AH ●) and combined treatment with silane (water (WSH ♦); alkali (ASH **+**)).

Figure 6a presents the measured and predicted adsorption isotherms for the neat PLA and the 30 wt.% reinforced composites while Figure 6b presents the outcome at 50% fiber loading. All samples displayed similar patterns in the form of sigmoidal shapes, classified as a type II isotherm, synonymous with cellulose-based materials [25]. Generally, the neat PLA remained significantly unaffected by the changing humidity conditions while the moisture adsorption decreases with fiber treatment and increased with the fiber contents. The highest EMC of 11.76% (at 95% RH) was displayed by UH (50 wt.%), which was significantly decreased by 18% following alkali pre-treatment and 16% with combined alkali and silane treatments. For 30 wt.% composites, there was no notable difference in the outcome for AH and ASH, though at 50 wt.%, a 13% meaningful reduction in EMC was achieved for ASH composites.

To complete Figure 6, Table 4 shows the difference of the results at 95% RH and the level of significance according to the ANOVA analysis. The most essential outcomes are by the chemically treated hemp fiber-reinforced composites, showing low *P*-values.

The significant composite moisture adsorption at 50 wt.% fiber loading (1.5 to 1.8 times the moisture absorption of the 30% fiber loading at 95% RH) is ascribable to two combined factors: (1) the presence of higher fibrous ratio with more hydrophilic groups than the PLA matrix, (2) the higher volume of porosities for the 50 wt.% fibers highlighted in the Section 3.2 (Table 3). According to Pejic et al. [14], the amount of free amorphous and crystalline OH available in hemp fiber determines moisture sorption up to a RH of 65%, whereas the biochemical compositions (lignin, pectin, hemicellulose, and celluloses) and their locations are the main factors influencing the moisture absorption by capillary condensation above the RH of 65%. Thus, the decreased EMC for the AH composite can be attributed to (i) less free OH groups, and (ii) a change in the fiber microstructure described by Alao et al. [10] and (iii) enhanced bonding between the fiber and PLA as demonstrated in Figure 5. The negligible effect of water fiber pretreatment on composite moisture adsorption compared to untreated fiber composites might be due to the moderate removal of the fiber hemicellulose content [10], as indicated by the low mass loss associated to this pretreatment (Table 2).

The values of the *A*, *B*, *C* parameters from the modified Oswin model with the calcu-lated standard error of estimate (E_S_) and the mean relative percentage deviation (Pd.), for all the composite specimens’ adsorption are presented in Table 5. To access the accuracy of this model, the Es and Pd. values are used. A model is reasonably accepted if the estimated Pd. is below 10% [26]. The calculated Pd. for all the composites did not exceed 10% while the Es were also low (<1), which confirms the suitability of this model in predicting the moisture adsorption of hemp fiber reinforced PLA composites. No study has been found using such model fitting to describe the isotherms of HPLA composites. However, Nilsson et al. [19] reported an Es and Pd. value of 0.68 and 4.19% respectively, for frost retted hemp fibers.

### 3.4. Water Absorption and Thickness Swelling Results

**Figure 7:**
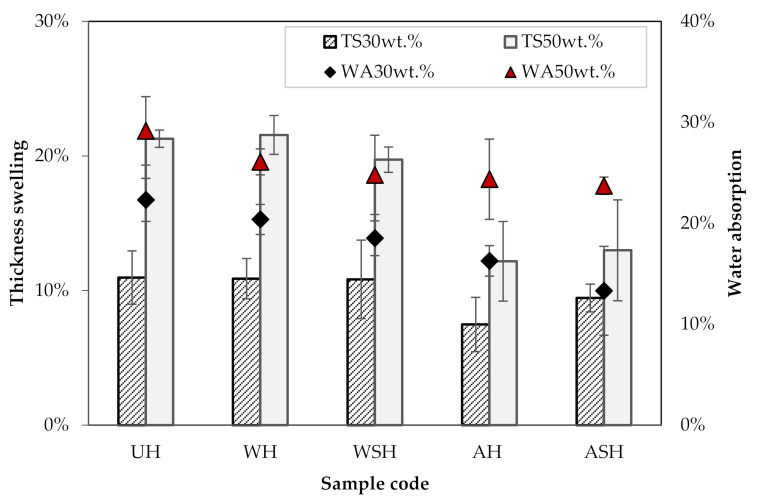
Water absorption and thickness swelling of the HPLA composites.

Figure 7 presents the water absorption (*WA*) and thickness swelling (*TS*) of the HPLA composites. The neat PLA showed no noticeable changes in mass or thickness (0%) after the 28 days of total immersion, so the associated values are not reported in the Figure 7. With the composites, the moisture affinity is the highest for the 50 wt.% fiber reinforced composite. The UH composite exhibited the most change in *WA* and *TS* of 23 and 48%, respectively. Compared to the UH, WH composites showed a slight reduction in WA, of 9% (30 wt.% fibers) and 11% (50 wt.% fibers), but the *TS* appeared unchanged. Nonetheless, compared to UH, the WSH composite *TS* for 50 wt.% fiber showed a significant decrease, and a notable reduction in WA for both fiber ratios. Compared to UH, AH composites showed a 27% drop in *WA* at 30 wt.% fiber content, and 16% drop at 50 wt.%, while the *WA* of ASH composites further decreased by 13 and 3% at 30 wt.% and 50 wt.% fibers, respectively. However, the difference between the outcomes for ASH and AH was statistically insignificant.

The low *WA* for AH and ASH composites can be ascribed to (i) the changes that have occurred to the porous structure of the hemp fibers in the case of the alkali pretreatment, (ii) the better interfacial polymer/fiber adhesion induced by the silane coupling and, (iii) the reduced hydrophilic properties arising from the less available OH needed for bonding with water molecules. Moreover, AH and ASH are the composites which contain the less porosities, at both 30 and 50 wt.%, thanks to the efficient removal of non-cellulosic components by the alkali treatment, and to the silane coupling that enhance the fiber-matrix interface. The reduced level of porosity also partially contributes to the low WA of these composites. When a strong bond exists between the polymer matrix and the fiber, better packing occurs in the composite, leading to a decrease in the distance travelled by the diffusion water molecules and a reduction in the composite’s water uptake [27]. Alkali pretreatment of hemp fibers changes the surface topology, inducing a higher roughness that leads to better fiber/matrix anchorage, and removed non-cellulosic impurities from the fiber surface, allowing and improving bonding with the matrix due to a better accessibility of hydroxyl groups of cellulose. Väisänen et al. [28] and Baghaei et al. [29] obtained results similar to that of this current study. Väisänen et al. [28] reported 30% WA after 28 days of immersion for the untreated hemp-epoxy composites that decreased to 12.5% *WA* following alkali treatment, while Baghaei et al. [29] showed a *WA* of about 21% for untreated, nonwoven HPLA composites after 10 days of water immersion. Conversely, Sgriccia et al. [30] showed that for 25 wt.% hemp/epoxy composites, the NaOH treatment prompted higher moisture absorption than the untreated fiber composites, which was related to the presence of voids induced by the rougher fiber surface following the treatment.

### 3.5. Reaction to Fire of Composite Materials

#### 3.5.1. Temperature Response through Depth

**Figure 8:**
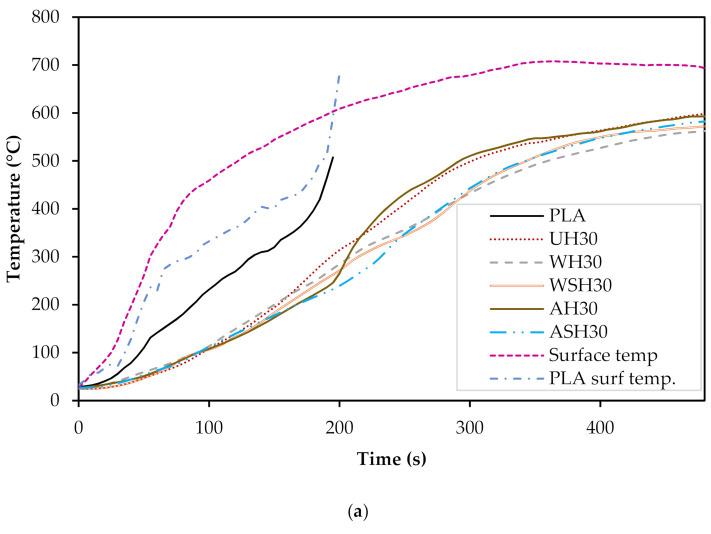

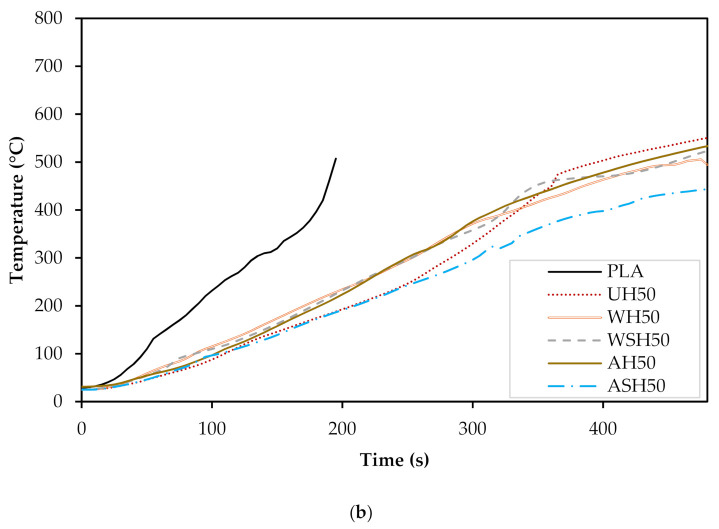
Surface temperature and temperature response behind the neat PLA and composites at (**a**) 30%;(**b**) 50% hemp fiber content.

The average surface temperatures and temperature response (i.e., temperature determined between the surface of the composite and the timber block) of neat PLA and the 30 wt.% composites are presented in Figure 8a, while Figure 8b shows the temperature response for the 50 wt.% composites. The average surface temperature rose from 27 to 693 °C, while the temperature response through depth measured between the surface of the composite and the timber was at a maximum, 598 °C and 550 °C for UH_30_ and UH_50_, respectively. The exposure to the heat flux was performed for 480 s. However, the neat PLA boards completely decomposed after about 180 s. Composites with 30 wt.% fibers displayed higher temperatures measured between the interface of the composite and the timber than composites with 50 wt.% fibers. For instance, for a surface temperature range of 300 to 400 °C (regarded as the temperature range for the pyrolysis of cellulose), UH_30_ produced an average of 61 °C temperature response through depth compared to UH_50_ (57 °C). The reaction to fire of the neat PLA was shown as a reference. Compared to the composites, the PLA has a rapid ignition (as measured from the temperature response through depth) and decomposition (180 s). It is thus observed that the 30 wt.% composites with higher PLA content, when compared to composites of 50 wt.% fibers, showed a slightly higher temperature response through depth and also higher rate of decomposition.

The Basic protection time (t_prot_) of the specimens and start of char of the timber base (t_ch_) did not present meaningful difference between the composites.

#### 3.5.2. Other Reaction to Fire Test Results

Table 6 displays the specimen thickness, density, mass prior to testing, mass loss after fire exposure, ignition time and ignition temperature. The average weight and thickness of the composites were greater for fiber reinforced PLA composites with 50% loading. The discrepancy in weight and thickness could be attributed to the bulky nature of hemp fiber and the density difference between PLA and fiber. In terms of ignition temperature and mass loss, there does not appear to be any significant improvement with the increase in fiber content, but the average mass loss was about 4% lower compared to the 30% fiber content, which is due to higher ash content at the higher fiber amount. The average temperature for AH and combined treatments (WSH and ASH) were significantly improved at 50 wt.% compared to 30 wt.%, but the changes for UH and WH were statistically insignificant. Ahmed et al. [31] also demonstrated that fiber modification by grafting of silane coupling agents can improve the thermal stability of the composites. Moreover, the ignition temperatures of UH and WH composites at both fiber compositions were identical, which might be attributed to the lack of a major difference in the fiber cell wall content. Although it is possible that the increased ignition temperature for WSH is due to the silane coupling agent, SEM pictures (Figure 5) and fiber mass change (Table 2) revealed inconsistencies in cell wall structure and composition after combine modification that implies that the better performance is the result of the extraction of portion of the fiber cell wall components and the silane molecules attached to the fiber surface.

When compared to the neat PLA, the ignition temperature of all the composites increased. The deviation observed in the ignition properties within series are attributable to the fabrication of the replica from separate boards rather than the machining of a single board. Furthermore, even though the cone heater was pre-calibrated before each test, the ambient conditions slightly influenced ignition properties since the experiments were performed over several days. The delayed composite ignition with increase in fiber volume is reportedly attributed to the higher density of the composite [16]. However, the current study’s findings demonstrate that, while ignition time rises with fiber content, there appears to be no association with composite densities. At 30%, there was no significant variation in the densities of the composites. When the density of the composites at the same fiber content was compared to the ignition time, it was discovered that though the 30 wt.% WH had the highest density, the ignition time difference (28%) was insignificant compared to AH, which had 3% less density. In addition, at 50 wt.%, despite the decreased density of WH compared to UH, the ignition time was dramatically delayed by 35%. The results of thermogravimetric analysis earlier published [10] found that hemp fiber surface pretreatments and treatments improved the fiber’s thermal stability because of the removal of non-cellulosic contents that have lower degradation temperature. This improvement of thermal stability at the microscale after treatment could be the main reason influencing the fire resistance of the hemp reinforced PLA composites. It may be summarized that the reaction to fire of hemp fiber reinforced PLA composites depends largely on the PLA/fiber content and fiber surface pretreatment, though a more detailed study may be required. 

## 4. Conclusions

In this research, the moisture, water resistance and response to fire performance of hemp fiber reinforced polylactide composites were investigated in regard to a combination of fiber surface treatments. Moisture behaviour and reaction to fire of hemp fiber reinforced PLA composites were found to largely depend on the PLA/fiber content and fiber surface (pre)treatment, though a more detailed study of the fire behaviour of the composite may be required. Besides improving the fiber dispersion and homogeneity within the composites, alkali pretreatment and silane modification were found to decrease the composite’s hydrophilic characteristics. Water pretreatment showed little effect on the composite studied characteristics; however, the further silane modification produced significantly higher moisture resistance and better reaction to fire, which may be attributed to the extra removal of the amorphous cell wall content and silane coupling at the hemp fiber surface. The Oswin model accurately predicted the adsorption isotherm for all composites.

Although composites containing higher hemp fiber content were more sensitive to moisture/water, a greater fire resistance was discovered, resulting in a slower rate of thermal breakdown owing to the low PLA ratio. The combined surface treatments of the fibers primarily improved the composite fire protection qualities. Overall, the alkali pretreatment of hemp fibers and surface modification with silane led to the most promising results for the use of hemp reinforced PLA composites. However, considering the expense of silane agents, a simple alkali treatment may be sufficient to efficiently increase the considered properties. This study showed that adequate hemp fiber surface treatment allows the improvement of the composite durability during service, which opens opportunities for the use of sustainable composites in the transportation and construction sectors.

Future study will examine the impact of fire-retardant treatment as a means to improve hemp fiber reinforced composite fire performance, the effect of the fiber surface pretreatment on the effectiveness of the fire-retardant modification and the overall impact of the combined fiber surface and fire-retardant treatment on the mechanical and physical properties of the composites.

## Figures and Tables

**Table 1:** The percentage biochemical composition of the untreated frost-retted hemp fibres used in this study [17].

Cellulose	Hemicellulose	Lignin	* Solubles	Inorganic Matter
77.4 ± 0.3	8.3 ± 0.3	1.4 ± 0.0	12.6 ± 0.4	0.3 ± 0.0

* Soluble content is characterized by pectin + wax + water extractives.

**Table 2:** The mass change of the hemp fiber following pretreatment/treatment.

Treatment	Weight Change %
Water	−4.0 ± 0.3
Alkali treatments	−14.2 ± 0.8
Silane modification of water pre-treated fibers	−3.0 ± 0.6
Silane modification of alkali pre-treated fibers	+0.9 ± 0.0

**Table 3:** Volume of porosities of the composites.

Composites	30 wt.% *V_P_* (%)	50 wt.% *V_P_* (%)
UH	10	13
WH	6	13
WSH	7	9
AH	4	7
ASH	4	8

**Table 4:** The differences between the *EMC* of the HPLA composites (30 and 50 wt.%) at 95% RH and the obtained *P*-value from an alpha of 0.05 (ANOVA single factor analysis).

	30 wt.% HF		50 wt.% HF	
Composite Comparison	Difference at 95% RH	*P*-Value	Difference at 95% RH	*P*-Value
WH < UH	6	0.144	5	0.5
AH < UH	18	0.012	14	0.012
WSH < UH	24	0.001 **	21	0.002
ASH < UH	16	0.001	25	0.001 **
WSH < WH	19	0.006	17	0.002
AH < ASH/AH > ASH	4	0.603	13	0.002
WSH < AH	7	0.369	4	0.034
WSH < ASH	10	0.051	5	0.144

** lesser than 0.001.

**Table 5:** Adsorption parameters and goodness of fit for Oswin model determined from sorption isotherms of untreated (UH), and treated (WH, WSH, AH and ASH) composites at 30 and 50 wt.% of hemp fiber content.

	Constants			ROOT Mean Square (R^2^)	Standard Error of Estimate (Es)	Pd., %
	*A*	*B*	*C*			
30 wt.% HF						
UH	1.48	0.02	2.22	0.997	0.17	7.2
WH	1.37	0.02	2.21	0.996	0.19	8.9
WSH	0.10	0.00	2.37	0.991	0.22	8.7
AH	0.95	0.01	1.97	0.998	0.12	8.0
ASH	1.30	0.02	2.33	0.995	0.19	8.8
50 wt.% HF						
UM	2.54	0.02	2.13	0.998	0.24	6.2
WH	2.29	0.02	2.06	0.987	0.57	5.7
WSH	2.13	0.02	2.25	0.997	0.22	6.6
AH	1.97	0.02	2.02	0.998	0.19	6.3
ASH	2.04	0.02	2.28	0.997	0.20	6.1

**Table 6:** Specimen properties, mass loss and ignition time and temperature.

Fiber Content	Samples	Weight of Board before Test (g)	Thickness (mm)	Density (gcm^−3^)	Average Ignition Time (s)	Ignition Temperature (°C)	Mass Loss (%)
30%	UH	28 ± 1.8	2.8 ± 0.4	1.01 ± 0.1	29 ± 10	112 ± 23	93 ± 2.0
	WH	27 ± 1.4	2.5 ± 0.2	1.10 ± 0.0	31 ± 10	117 ± 18	95 ± 0.1
	WSH	28 ± 1.2	2.6 ± 0.3	1.04 ± 0.1	30 ± 04	131 ± 28	94 ± 0.4
	AH	26 ± 0.6	2.5 ± 0.2	1.07 ± 0.1	44 ± 02	118 ± 08	94 ± 1.0
	ASH	28 ± 1.6	2.7 ± 0.2	1.07 ± 0.0	33 ± 03	159 ± 18	94 ± 2.0
50%	UH	29 ± 0.8	3.2 ± 0.2	0.91 ± 0.1	32 ± 07	129 ± 15	90 ± 1.0
	WH	29 ± 1.8	3.3 ± 0.2	0.90 ± 0.1	49 ± 06	134 ± 17	91 ± 2.0
	WSH	30 ± 1.0	3.1 ± 0.1	0.97 ± 0.0	46 ± 10	179 ± 29	90 ± 1.0
	AH	30 ± 0.3	3.2 ± 0.1	0.92 ± 0.0	51 ± 10	178 ± 18	90 ± 4.0
	ASH	30 ± 0.6	3.2 ± 0.1	0.98 ± 0.0	47 ± 09	181 ± 18	89 ± 1.0
0%	Neat PLA	26 ± 1.2	2.2 ± 0.2	1.2 ± 0.1	37 ± 02	104 ± 04	100 ± 0.0

## Data Availability

Data sharing not applicable.
